# Soil metagenomics reveals the effect of nitrogen on soil microbial communities and nitrogen-cycle functional genes in the rhizosphere of *Panax ginseng*

**DOI:** 10.3389/fpls.2024.1411073

**Published:** 2024-08-07

**Authors:** Kexin Li, Hongmei Lin, Mei Han, Limin Yang

**Affiliations:** College of Traditional Chinese Medicine, Jilin Agricultural University, Changchun, China

**Keywords:** ginseng, rhizosphere soil, metagenomics, microbial composition, nitrogen functional genes

## Abstract

Nitrogen (N) is the primary essential nutrient for ginseng growth, and a reasonable nitrogen application strategy is vital for maintaining the stability of soil microbial functional communities. However, how microbial-mediated functional genes involved in nitrogen cycling in the ginseng rhizosphere respond to nitrogen addition is largely unknown. In this study, metagenomic technology was used to study the effects of different nitrogen additions (N0: 0, N1: 20, N2: 40 N g/m^2^) on the microbial community and functional nitrogen cycling genes in the rhizosphere soil of ginseng, and soil properties related to the observed changes were evaluated. The results showed that N1 significantly increased the soil nutrient content compared to N0, and the N1 ginseng yield was the highest (29.90% and 38.05% higher than of N0 and N2, respectively). N2 significantly decreased the soil NO_3_^–^N content (17.18 mg/kg lower than N0) and pH. This resulted in a decrease in the diversity of soil microorganisms, a decrease in beneficial bacteria, an increase in the number of pathogenic microorganisms, and an significant increase in the total abundance of denitrification, assimilatory nitrogen reduction, and dissimilatory nitrogen reduction genes, as well as the abundance of *nxrA* and *napA* genes (17.70% and 65.25% higher than N0, respectively), which are functional genes involved in nitrification that promote the soil nitrogen cycling process, and reduce the yield of ginseng. The results of the correlation analysis showed that pH was correlated with changes in the soil microbial community, and the contents of soil total nitrogen (TN), ammonium nitrogen (NH_4_^+^-N), and alkaline-hydrolyzed nitrogen (AHN) were the main driving factors affecting the changes in nitrogen cycling functional genes in the rhizosphere soil of ginseng. In summary, nitrogen addition affects ginseng yield through changes in soil chemistry, nitrogen cycling processes, and functional microorganisms.

## Introduction

Nitrogen (N) is the primary nutrient required for plant growth (Xia et al., 2023). Nitrogen deficiency can lead to dwarfism, slow plant growth, and reduced chlorophyll content, photosynthesis and plant productivity. Excessive nitrogen input exceeds the nitrogen demand of plants and soil microorganisms, which is not only beneficial for increasing yield, but will also reduce nitrogen utilization efficiency (Sung et al., 2023). A large amount of surplus nitrogen is easily lost through runoff, leaching, ammonia volatilization, and denitrification. It will further cause negative ecological effects such as soil acidification, biodiversity reduction, water eutrophication, and greenhouse effects, adversely affecting the biogeochemical cycle of agroecosystems (Erisman et al., 2013; Rosemond et al., 2015; Sun et al., 2021; Wang X. et al., 2023). Soil nitrogen cycling is one of the most important processes in agroecosystems and mainly includes nitrogen fixation (NF), nitrification (Nit), denitrification (Den), assimilatory nitrogen reduction (ANR), and dissimilatory nitrogen reduction and (DNR). Microorganisms drive the entire nitrogen cycle process (Kuypers et al., 2018). With advancements in research technology, researchers have studied soil nitrogen cycling more deeply, from changes in microorganisms to related functional genes. Metagenomic technology has been effectively applied to studying functional genes related to soil nitrogen cycling, which can more comprehensively detect multiple genes involved in soil nitrogen cycling processes and help accurately evaluate the response of microbially driven changes in soil nitrogen cycling processes to nitrogen addition (Delmont et al., 2011; Thamdrup, 2012; Han et al., 2021; Ku et al., 2022).

Certain studies have shown that nitrogen addition can lead to changes in the abundance and composition of soil nitrogen cycling functional genes (Scarlett et al., 2021; Fudjoe et al., 2023). In the North China Plain, 20 years of field experiments showed that long-term nitrogen addition increased the abundance of microorganisms involved in most of the nitrogen conversion processes, but decreased the abundance of nitrogen fixed combinations. Although there were no significant changes in the abundance of several functional genes, the composition of the microbiota involved in each nitrogen conversion process was altered by nitrogen application (Sun et al., 2021). There were significant differences in the structure and composition of the bacterial communities in the rhizosphere of *Lycium barbarum* under different nitrogen supply conditions, and high nitrogen addition inhibited the diversity and stability of the bacterial communities. Ammonia oxidizing bacteria (AOB) are important participant in the soil nitrogen cycle, catalyzing the first step in the ammonia oxidation process. *Nitrosospira* is an important species of AOB, and low nitrogen input stimulates the increasing the *Nitrosospira* relative of *Nitrosospira* sp. (Li Y. et al., 2022). In addition, high nitrogen addition significantly increased the expression of the *amoB* genes involved in ammonia oxidation but did not significantly affect the abundance of denitrifying *nirS* and *nirK* genes (Tian et al., 2014). However, it also led to a decrease in the abundance of *nifH*, *nirS* and *nosZ* owing to increased soil acidification (Ning et al., 2015).

*Panax ginseng* (*Panax ginseng* C.A.Mey.) is a perennial herb belonging to the family *Eleutheroaceae* and has high medicinal and economic value (Ma et al., 2024). In recent years, with the development of the traditional Chinese medicine in China, people’s demand for ginseng has led to an increase in ginseng production and efficiency year by year. However, the excessive application of chemical fertilizers and other reasons have caused the deterioration of soil properties and the change of microbial diversity and community structure. This ultimately leads to the decline of ginseng yield and quality. Among them, the factors affecting the diversity of microbial communities in the rhizosphere soil of ginseng have been divided into two categories: natural factors and human factors. Natural factors include soil type, seasonal changes (temperature and moisture), and pH, whereas anthropogenic factors include years of planting, farming practices, and fertilization. Fertilization mainly affects plant growth indirectly by altering the physical and chemical properties of the soil and soil microbial diversity (Yang et al., 2020; Lan et al., 2023; Huang et al., 2024). However, nitrogen is the main nutrient element for ginseng growth, and a reasonable nitrogen application strategy is very important to maintain the stability of the soil microbial functional community and improve soil fertility and yield (Gong et al., 2023). Yet, little attention has been paid to its effects on soil properties, especially on the changes of rhizosphere microbial communities, and how the rhizosphere soil environment of ginseng changes the rhizosphere microbiome and affects the microbial-driven soil nitrogen cycling process under different nitrogen levels is still unknown.

Therefore, we determined the yield and rhizosphere soil chemistry of ginseng under different nitrogen levels in this study. Metagenomic technology was used to comprehensively analyze the differences in microbial communities and nitrogen cycling functional genes in the rhizosphere soil of ginseng under different nitrogen levels. The objectives of this study were: (1) to explore the effects of nitrogen on the chemical properties and microbial communities of ginseng rhizosphere soil; (2) to elucidate the effects of nitrogen on microbial functional genes in different processes of soil nitrogen cycling; (3) to reveal the main driving factors affecting microbial communities and functional genes in ginseng rhizosphere soil at different nitrogen levels.

## Materials and methods

### Sample collection and site description

The test site was located at the base of Jilin Province Shenwang Plant Protection Company (Xiaoshan Village, Songjianghe Town, Baishan City, Jilin Province, 127°23’30’’E and 42°13’26’’N, 727.8 masl), The altitude was 727.8 m, and the air pressure was 929.4 hPa. The average annual temperature is 3.7 °C, and the average annual precipitation is 712.5 mm. The chemical properties of the tested soil were pH 5.28, the soil total nitrogen (TN) 1.54g/kg, the soil total phosphorus (TP) 0.68g/kg, the soil total potassium (TK) 19.51g/kg, the soil alkaline-hydrolyzed nitrogen (AHN) 119.19 mg/kg, the soil available phosphorus (AP) 18.23mg/kg and total potassium (AK) 90.18 mg/kg.

Three nitrogen treatments: 0 (N0), 20 (N1) and 40 (N2) N g/m^2^ were set in the ginseng cultivation soil, and the application rates of phosphate (P) and potassium (K) were 40 g/m^2^ and a random block design was adopted, with each treatment being 5 m^2^ and repeated three times, a total of 9 plots (among them, the 0 N and 40 N treatments were the lowest and highest nitrogen application rates, and the 20 N treatment was the optimal agronomic nitrogen application rate recommended by soil chemistry and local planting experience before treatment). Fertilizer (N: 46% urea, P: 12% superphosphate, K: 50% potassium sulfate) was applied as a basal fertilizer to the experimental field at one time on April 20, 2022. Disease-free, uniformly sized 2-year-old ginseng seedlings were selected for transplanting on April 21, 2022, and field management such as weeding and insecticides was carried out regularly. The sampling was conducted on September 20, 2022, during the ginseng harvest period. Soil sampling was carried out by random sampling method, and the rhizosphere soil attached to the roots of ginseng was about 2 mm thick with a brush in each treatment group. The soil was screened through a 2 mm mesh sieve after mixing, the soil organic matter (OM) needs to pass through a 0.147 mm mesh sieve, and the roots were removed and divided into three parts. These were placed in a cool place to be naturally air-dried for soil property determination, at 4°C for the soil ammonium nitrogen (NH_4_^+^-N) and the soil nitrate nitrogen (NO_3_^–^N) determination, and divided into cryopreservation tubes and placed in a −80°C freezer after liquid nitrogen flash-freezing for metagenomic sequencing.

### Determination of soil chemistry and ginseng yield

The soil pH value was determined using an international standard 1:5 soil-water ratio while using an ion meter (MP521, Sanxin Instrument Factory, Shanghai, China); the OM content was determined using a dilution heat method with potassium dichromate; the TN was determined using Kjeldahl nitrogen determination (K9860, Haineng Scientific Instrument Co., Ltd., Shandong, China); the AHN content was determined using an alkaline-hydrolyzed diffusion method; the TP was determined using the NaOH melt-molybdenum-antimony anticolorimetric method; the AP content was determined by a sodium bicarbonate extraction and molybdenum-antimony resistance colorimetric method; the AK and TK were determined using flame atomic absorption spectrophotometry (AA-6300, Shimadzu Corporation, Japan) (Bao, 2000); the NH_4_^+^-N was determined using the NaCl colorimetric method; the NO_3_^–^N was determined using NaCl spectrophotometry (Cui and Jiang, 2020). The ginseng yield was calculated as follows: 2.5 m^2^ sample was taken in each treated field, and all ginseng plant weights were accurately weighed with an electronic balance, yield (g/m^2^)=the sum of all individual plant weights (g)/2.5.

### Soil DNA extraction and metagenomic sequencing

DNA from different samples was extracted using CTAB according to manufacturer’s instructions. The reagent which was designed to uncover DNA from trace amounts of sample has been shown to be effective for the preparation of DNA of most bacteria. Sample blanks consisted of unused swabs processed through DNA extraction and tested to contain no DNA amplicons. The total DNA was eluted in 50 µl of Elution buffer by a modification of the procedure described by manufacturer (QIAGEN)and stored at -80°C until measurement in the PCR by LC-BIO (TECHNOLOGIES (HANGZHOU) CO., LTD., Hang Zhou, Zhejiang Province, China). DNA library was constructed by TruSeq Nano DNA LT Library Preparation Kit (FC-121-4001). DNA was fragmented by dsDNA Fragmentase (NEB, M0348S) by incubate at 37°C for 30 min. Library construction begins with fragmented cDNA. Blunt-end DNA fragments are generated using a combination of fill-in reactions and exonuclease activity, and size selection is performed with provided sample purification beads. An A-base is then added to the blunt ends of each strand, preparing them for ligation to the indexed adapters. Each adapter contains a T-base overhang for ligating the adapter to the A-tailed fragmented DNA. These adapters contain the full complement of sequencing primer hybridization sites for single, paired-end, and indexed reads. Single- or dual index adapters are ligated to the fragments and the ligated products are amplified with PCR by the following conditions: initial denaturation at 95°C for 3 min; 8 cycles of denaturation at 98°C for 15 sec, annealing at 60°C for 15 sec, and extension at 72°C for 30 sec; and then final extension at 72°C for 5 min.

Raw sequencing reads were processed to obtain valid reads for further analysis. First, sequencing adapters were removed from sequencing reads using cutadapt v1.9. Secondly, low quality reads were trimmed by fqtrim v0.94 using a sliding-window algorithm. Thirdly, reads were aligned to the host genome using bowtie 2v2.2.0 to remove host contamination. Once quality-filtered reads were obtained, they were *de novo* assembled to construct the metagenome for each sample by MEGAHIT v1.2.9 (**Supplementary Table S1**). All coding regions (CDS) of metagenomic contigs were predicted by MetaGeneMark v3.26.CDS sequences of all samples were clustered by CD-HIT v4.6.1 to obtain unigenes. Unigene abundance for a certain sample were estimated by TPM based on the number of aligned reads by bowtie 2v2.2.0. The lowest common ancestor taxonomy of unigenes were obtained by aligning them against the NCBI NR database by DIAMONDv0.9.14. Similarly, the functional annotation of unigenes were obtained. Based on the taxonomic and functional annotation of unigenes, along with the abundance profile of unigenes, the differential analysis were carried out at each taxonomic or functional or gene-wise level by Fisher’s exact test (non-replicated groups) or Kruskal-Wallis test (replicated groups). The aligned putative amino acid sequences were annotated based on the Kyoto Encyclopedia of Genes and Genomes database (KEGG) using BLAST (version 2.2.21). Gene families involved in the nitrogen cycle were selected from the KEGG database (**Supplementary Table S2**). TPM (transcripts per kilobase per million mapped reads) was used to normalize the abundance values in metagenomes for comparison between metagenomes of different sizes and averaged among replicate samples to assess the abundance of these genes. Fresh soil (0.15 g) was weighed, DNA was extracted using the Soil Genomic DNA Extraction Kit (Solarbio, Beijing), and qRT-PCR was performed using a real-time PCR instrument (Mx3000P, Aligent, America) to analyze the content of seven nitrogen cycle genes, including *amoA*, to validate the metagenomic data. The qPCR reaction was 20 μL and comprised 1 μL of DNA template, 10 μL of SYBR^®^ Premix Ex Taq™, 1 μL of forward and reverse primers, 1 μL of DNA template, and 7 μL of ddH_2_O. Primer sequences for the nitrogen cycle functional gene are listed in **Supplementary Table S2**.

### Statistical analysis

The differences in ginseng yield, rhizosphere soil chemistry and the abundances of microbial functional genes among the treatments were analyzed using SPSS 27.0 software. GraphPad Prism 8.0.2 was used to plot the data. Shannon and evenness indices were calculated for alpha diversity using the “vegan” package in R and then compared among treatments using the Kruskal-Wallis test. The principal coordinate analysis (PCoA) was performed on Bray-Curtis dissimilarities using the “vegan” package to determine the compositional differences among treatments and compartments. Dissimilarities within treatments were also calculated. Relationships between the abundances of microbial functional gene groups that involved in soil nitrogen cycling and soil properties were determined using the Mantel test and Pearson’s correlation. The relationships between microbial composition and soil properties were determined using the correlation network analysis. These analyses were performed using R-3.4.4 software.

## Results

### Soil chemical properties and crop yield

The addition of N significantly altered the rhizosphere soil chemistry of ginseng (**Table 1**). We found that the levels of OM, TN, TP, TK, AHN, AP, AK, and NH_4_^+^-N in the soil increased significantly with increasing nitrogen concentrations (*p*<0.05), compared with N0, the contents of AHN in the nitrogen addition treatment group (N1 and N2) increased by 7.82% and 26.99%, respectively, and the content of AK increased by 1.36 times and 1.56 times, respectively. TN and TK in N2 increased by 0.50 g/kg and 13.32 g/kg compared with N0, respectively, and the contents of TP and AP increased significantly in the N1 (0.27 g/kg and 3.94 g/kg, respectively) and N2 (0.31 g/kg and 4.29 mg/kg, respectively) treatment groups. Compared with N0, the content of NH_4_^+^-N in the N1 and N2 treatment groups increased by 22.06% and 29.14%, respectively. With the increase in nitrogen concentration, and the OM in the N2 treatment increased by 16.86% compared with that in N0, and NO_3_^–^N in the soil showed a trend of first increasing and then decreasing, while the pH value decreased significantly with the increase in nitrogen concentration. Compared with N0, the NO_3_^–^N content in the N1 treatment increased by 19.32 mg/kg, and the NO_3_^–^N content in the N2 treatment decreased by 17.18 mg/kg. In conclusion, appropriate nitrogen addition can increase soil nutrient content; however, excess nitrogen can significantly reduce soil NO_3_^–^N content and soil pH.

**Table 1:** Relationship between soil chemical properties and ginseng yield under different nitrogen levels.

Treatment	pH	OM %	TN g/kg	TP g/kg	TK g/kg	AHN mg/kg	AP mg/kg	AK mg/kg	NH_4_^+^-N mg/kg	NO_3_^–^N mg/kg	Yield g/m^2^
N0	5.23 ± 0.03a	2.27 ± 0.02b	2.12 ± 0.02b	0.85 ± 0.02b	20.43 ± 0.05b	138.29 ± 5.83c	32.79 ± 1.36b	120. 20 ± 5.68c	31.12 ± 1.43c	105.39 ± 3.70b	628.59 ± 33.22b
N1	5.20 ± 0.02ab	2.37 ± 0.08b	2.14 ± 0.02b	1.12 ± 0.05a	20.77 ± 0.59b	149.11 ± 3.27b	36.73 ± 1.57a	163.81 ± 5.65b	39.93 ± 1.49b	124.71 ± 4.42a	816.56 ± 31.76a
N2	5.18 ± 0.01b	2.55 ± 0.06a	2.62 ± 0.06a	1.16 ± 0.06a	21.75 ± 0.40a	175.62 ± 2.80a	37.08 ± 0.35a	187.34 ± 8.49a	56.35 ± 0.68a	87.61 ± 2.13c	591.49 ± 38.42b

The letters “a, b and c” are significant markers, and different letters between treatments indicate significant differences at the significant level of 0.05.

The ginseng yield first increased and then decreased with an increase in nitrogen concentration; the yield of the N1 group was the highest, reaching 816.56 g/m^2^, which was 29.90% and 38.05% higher than that of the N0 and N2 groups, respectively. This indicated that nitrogen concentrations that were too low or too high would reduce the yield of ginseng, and the most suitable nitrogen concentration for ginseng growth in this experiment was 20 g/m^2^.

### Changes in soil microbial community diversity

**Figure 1:**
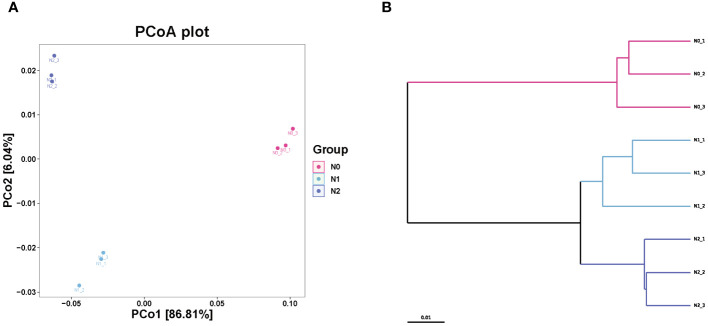
Effects of nitrogen on soil microbial community beta diversity. PCoA of microbial communities based on Bray-Curtis differences, Anosim: R^2 =^ 0.92 p=0.01 **(A)**; UPGMA hierarchical cluster analysis **(B)**.

All samples were sequenced, and the number of species found was 18,344-18,404 (**Supplementary Table S3**). Nitrogen addition did not significantly affect the number of observed species or goods coverage of the ginseng rhizosphere soil microbial community (*P* > 0.05). Compared with the non-nitrogen N0 group, the nitrogen treatment groups (N1 group and N2 group) showed significant increases in the richness index of Chao1, however, the diversity of Shannon and Simpson decreased with the decreasing nitrogen concentration, indicating that nitrogen addition increased the abundance of the soil microbial community but decreased its diversity (*P* < 0.05). The UPGMA method was used to perform a hierarchical clustering analysis of all samples (**Figure 1B**). The results showed that the N0 community structure was significantly different from that of the N1 and N2 treatment groups. PCoA analysis based on the Bray-Curtis distance matrix revealed significant differences in microbial communities among the N0 and N1 and N2 treatment groups, indicating that nitrogen greatly influenced the microbial community structure (**Figure 1A**).

### Changes in soil microbial community composition

A total of 5 kingdoms, 170 phyla, 302 classes, 516 orders, 1,019 families, 3,264 genera, and 19,229 species were found in the annotation of the total microbial species in the rhizosphere soil samples of ginseng from the three groups. Species annotation information was obtained by comparison with the NR database (**Supplementary Table S4** and **Supplementary Figure S1**), and at the highest taxonomic boundary, the microbial community composition of bacteria was dominant in each treatment. The relative abundances of bacteria in the N0, N1, and N2 treatment groups were 72.55%, 63.55%, and 63.12%, respectively; the relative abundances of fungi, archaea and viruses were 0.29-0.39%, 0.11-0.13%, and 0.10-0.12%, respectively; and the remainder were unannotated and classified. Overall, the proportion of bacteria in the N1 and N2 treatment groups decreased compared to that in the N0 group, whereas the proportion of fungi and viruses increased.

**Figure 2:**
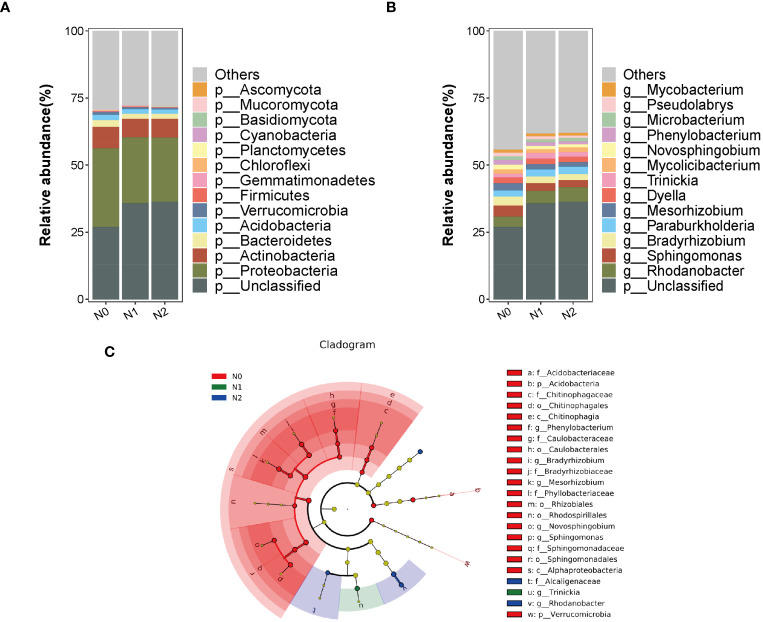
Distribution of the top 15 phyla **(A)** and genus **(B)** of soil microorganisms under different nitrogen levels. Analysis of multilevel species differences of soil microorganisms under different nitrogen levels **(C)**.

We selected 15 dominant bacteria with the highest relative abundance at the phylum and genus levels for analysis to investigate the effects of nitrogen changes on the microbial abundance and composition of the ginseng rhizosphere soil further (**Figures 2A, B**). Among them, Proteobacteria had the highest relative abundance, with the average relative abundance of N0, N1 and N2 treatments being 29.34%, 24.52% and 23.90%, respectively. This was followed by Actinobacteriota (7.86, 6.83 and 7.03%), Bacteroidota (2.51, 1.84 and 1.85%) and Acidobacteriota (2.06, 1.76 and 1.62%), with abundances higher than 1%; the relative abundance of Firmicutes and Ascomycota was significantly increased by in the N2 treatment, and the relative abundance of Basidiomycota was significantly increased by in the N1 and N2 treatments. The abundances of the other nine phyla decreased, except Mucoromycota, which was significantly reduced (*P<*0.05) (**Figure 2A**). The results indicated that the increase of nitrogen would reduce the abundance of bacterial microorganisms and increase the abundance of fungal microorganisms, and the beneficial bacteria would increase when the nitrogen level was too high to maintain the balance of soil microbial communities. The N1 and N2 treatments increased the relative abundance of *Rhodanobacter* (16.11% and 30.78%, respectively) and *Trinickia* (32.85% and 20.57%, respectively), compared with N0, but significantly decreased the abundance of *Sphingomonas*, *Bradyrhizobium*, *Mesorhizobium*, *Novosphingobium* and *Phenylobacterium* and *Microbacterium* (*P<*0.05) (**Figure 2B**).

### Differential analysis of LEfSe in soil microbial communities

LDA Effect Size (LEfSe) was used to analyze the changes in the composition of different species under different nitrogen levels (LDA>3.0, *P<*0.05). The results revealed significant differences in the 25 bacterial communities. There were 20 biomarkers in N0, 1 in N1, and 4 in N2 (**Figure 2C**). The N0 treatment was significantly enriched in Alphaproteobacteria, Caulobacteraceae, Bradyrhizobiaceae, Sphingomonadaceae, Phyllobacteriaceae*, Mesorhizobium*, *Novosphingobium*, *Bradyrhizobium*, *Phenylobacterium* and *Sphingomonas*, among which Proteobacteria are the most abundant. The genus *Trinickia* was significantly enriched in the N1 treatment, whereas *Alcaligenaceae*, *Rhodanobacte*, *Rhodanobacter_sp:C06* and *Mycolicibacterium_sp:P9_64* were significantly enriched in the N2 treatment.

### Analysis of functional genes related to soil nitrogen cycling

**Figure 3:**
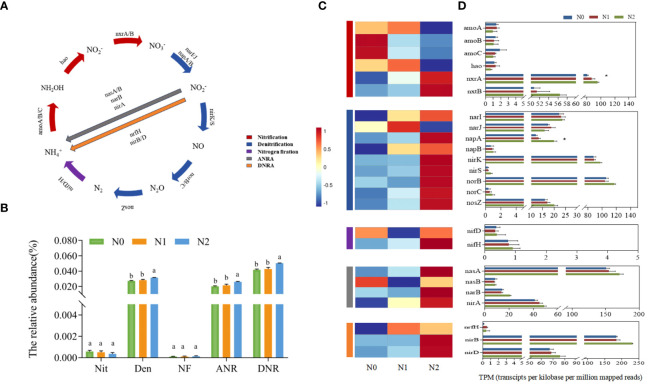
Differences in the abundance of gene families involved in N cycling functional genes under different nitrogen levels. N pathways considered in this study **(A)**. The total abundance of functional genes in the 5 and N cycle pathways under different nitrogen levels **(B)**. Nit, nitrification; Den, denitrification; NF, nitrogen fixation; ANR, assimilatory nitrogen reduction; DNR, dissimilatory nitrogen reduction; Heatmap of normalized abundance values (Row Z score) showing the difference in the abundance of N cycling functional genes in the KEGG database under different nitrogen levels **(C)**. Bars represented the means of standard errors **(D)** (replicates n=3). The total number of genes in each sample was normalized by the number of transcripts per kilobase per million mapped reads (TPM). Significant differences are marked by asterisks using Welch’s t-test in aldex2 analysis, *P < 0.05.

The metagenomics results showed that the total abundance of nitrogen cycle functional genes was significantly different under different nitrogen levels, with the N2 treatment having the highest abundance (**Figure 3B**). Among these five pathways, the gene abundance of the DNR the highest, whereas that of the NF was the lowest. Except for the fact that there was no significant difference between Nit and NF, the total abundance of genes involved in other nitrogen pathways in N2 treatment was significantly higher than that in N0 and N1 treatments. Nitrogen addition significantly changed the abundance of individual genes in the rhizosphere soil of ginseng (**Figure 3D**). The relative abundance of Nit genes in the three groups was the highest, and the relative abundance of other gene involved in Nit (*amoA*, *amoB*, *amoC* and *hao* genes) were lower than those of the five normalized reads. The N1 and N2 treatments increased the relative abundance of *nxrA* and *nxrB* genes, while decreasing that of *amoA*, *amoB*, *amoC*, and *hao* genes. The relative abundances of *narI*, *napA*, *napB*, *nirK*, *nirS*, *norB*, *norC* and *nosZ* Den genes in the N1 and N2 treatments were higher than those in N0. Only the *narJ* gene was lower in the N1 and N2 treatments than in N0, whereas the relative abundance of most genes involving ANR and DNR in the N1 and N2 treatments were higher than that in N0. Similarly, the relative abundance of the NF gene *nifH* in the soil of the N0 group was higher than that in the N1 and N2 treatments, but there was no significant change in *nifD*. These five pathways involved 24 genes, and *nxrA* and *napA* were significantly different among the three groups (**Figure 3D**). However, there was no significant effect on the total relative abundance of the Nit process (**Figure 3B**), indicating that nitrogen had a great influence on the DEN process, which was verified by the increase in Firmicutes. We also analyzed eight other genes involved in the nitrogen cycle, with the most abundant and significantly differentiated genes mainly involved in the glutamate synthesis pathway (Glu) (**Supplementary Table S5**). Seven representative and relatively abundant nitrogen cycle functional genes were selected for qRT-PCR analysis to verify the accuracy of metagenomic sequencing data (**Supplementary Figure S3**). The qRT-PCR results in the three treatments were consistent with the TPM trend, indicating that the metagenomic sequencing results were reliable.

### Relationship between soil nitrogen cycle functional genes and soil properties

**Figure 4:**
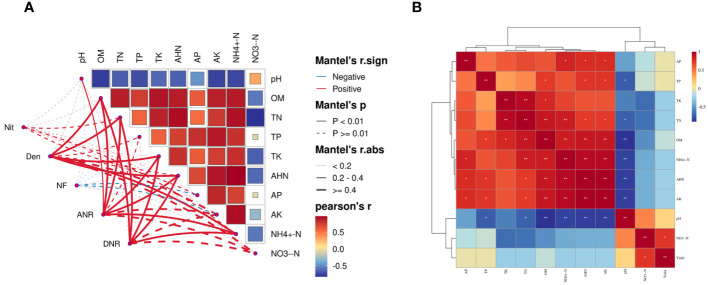
Relationship between functional genes of N cycle pathway and soil properties under different nitrogen levels **(A)**, (*p <*0.01). Relationship between ginseng yield and soil properties under different nitrogen levels**(B)**. Edge width corresponded to Mantel’s r-value, and edge color indicated statistical significance. Pairwise correlations of these variables are shown by color gradients representing Spearman’s correlation coefficients.

The Mantel test showed that the abundance of functional genes involved in Nit was significantly positively correlated with the content of soil TN and NH_4_^+^-N (*p*<0.05)(**Supplementary Figure S3**). Similarly, the abundance of functional genes involved in the Den process was significantly positively correlated with soil TN, NH_4_^+^-N, AHN, TK and OM content (*p*<0.01) (**Figure 4A**). There were no significant correlations between the abundance of functional genes involved in NF, and soil pH, OM, and soil N, P and K nutrients. TK, TN, OM, AHN and pH were significantly positively correlated with the abundance of functional genes involved in ANR (*p*<0.01). A significant positive correlation was observed between the abundance of functional genes of TN, NH_4_^+^-N, AHN, TK and OM functional genes in the soil involved in DNR (*p*<0.01). In summary, TN, NH_4_^+^-N and AHN were the main driving factors for the changes in nitrogen cycling genes in the ginseng rhizosphere soil.

## Discussion

Soil chemistry is a determining factor in soil fertility (Reichert et al., 2023). Appropriate nitrogen addition can effectively increase soil nutrient content, improve soil fertility, and further affect microbial changes, thereby increasing crop yield. However, nitrogen excess may reduce soil nutrients, destroy soil microbial community structure and diversity, and ultimately lead to a decrease in crop yield (Liu et al., 2020; Ruochen et al., 2021). The results of this study showed that soil organic matter content and the main nutrients (N, P, K) increased after nitrogen treatment. The OM, TN and TK contents in the N2 treatment were significantly higher than those in the N0 treatment, the contents of TP, AHN, AP and NH_4_^+^-N in the N1 and N2 treatments were significantly higher than those in the N0 treatment, and the contents of NO_3_^–^N in the N1 treatment were significantly higher than those in the N0 and N2 treatments (*P*<0.05). These results indicated that appropriate nitrogen addition could improve soil fertility and create a good environment for soil microbial communities, thereby promoting soil nutrient turnover. The increase of nitrogen concentration significantly reduced the soil pH (*P*<0.05). The pH value of the N2 treatment was the lowest, and the NO_3_^–^N concentration in the N2 treatment was significantly lower than in the N0 and N1 treatments. The correlation results indicated a significant positive correlation between ginseng yield and NO_3_^–^N (**Figure 4B**), indicating that excessive nitrogen addition would lead to the supply of nitrogen exceeding the nutrient requirements of soil microorganisms and ginseng, resulting in the loss of NO_3_^–^N in the soil through leaching and Den. This results in the production of a large amount of H^+^ in the soil, accelerating the process of soil acidification and reducing the yield of ginseng (Du et al., 2016; Wu et al., 2021).

Soil microorganisms are an important part of farmland ecosystems and play important roles in the mineralization, turnover, and uptake of soil nutrients (Hartmann and Six, 2022). Nitrogen addition directly affects the number and diversity of soil microorganisms owing to changes in soil pH and physicochemical properties (Li et al., 2018; Craig et al., 2021). The results of this study showed significant differences in soil microbial alpha and beta diversities (*P*<0.05), and the differences between the N1 and N2 treatments were not as large as those between the N1 and N0 treatments. However, nitrogen addition did not significantly affect the number of microbial community species in the ginseng rhizosphere soil. Nitrogen addition (N1 and N2) significantly increased the Chao1 richness index, but decreased the diversity of Shannon, and Simpson (*P*<0.05), Spearman’s correlation results showed that soil microbial diversity was significantly positively correlated with pH (*P*<0.01) (**Supplementary Figure S2**), and negatively correlated with OM, AHN and AK (*P*<0.01), indicating that the decrease in soil microbial diversity was closely related to soil acidification after nitrogen addition. This is consistent with the experimental results obtained by Dai (Dai et al., 2018). Nitrogen addition affected the diversity of microbial communities, and the composition of soil microbial communities (Yu et al., 2021; Zhou et al., 2022). There were significant differences in the soil microbial communities under different nitrogen levels, indicating that nitrogen affects the composition of soil microbial communities in the ginseng rhizosphere. Previous studies reported that Proteobacteria, Actinomycetes, Bacteroidetes, and Acidobacteria are the major microbial phyla in terrestrial ecosystems, which is consistent with our findings (Scarlett et al., 2021; Lin et al., 2022). Under different nitrogen levels in the three groups, the abundance of nine phyla, including Proteobacteria and Actinomycetes, decreased significantly with the increase in nitrogen addition, which led to differences in soil microbial community composition, mainly due to the different sensitivities and adaptation ranges of different types of microorganisms to environmental changes. Proteobacteria, Actinomycetes and Acidobacteria belong to a typical oligotrophic microbial group, which has a high nutrient affinity and strong adaptability to environments with low nutrient availability. Therefore, the low-nutrient N0 treatment showed a high relative abundance, which gradually decreased with increasing of nitrogen addition, which is similar to the results of previous studies (Zhang C. et al., 2019; Li H. et al., 2021; Qiu et al., 2021; Zhou et al., 2021). Studies have shown that *Planctomycetes* has a high affinity for ammonia nitrogen, which is suitable for slow growth in a dynamic environment with low ammonia oligotrophic nutrition, whereas a high ammonia and nutrient-rich environment is not conducive to survival, and belongs to the K-strategy microorganisms (Yun, 2022). In this study, the available nutrients in the soil increased, although NH_4_^+^-N increased significantly with an increase in nitrogen addition and the content of other nutrients; therefore, it was unsuitable for the growth of Planctomycetes, resulting in a gradual decrease in its abundance. However, the eutrophic organism Firmicutes, which responded positively to nitrogen addition, belongs to the R-strategy microorganisms, and the content of N2 was the highest. Some studies have shown that Firmicutes can promote soil fertility and plant growth, and participate in NF and Den in soil nitrogen cycle processes (Wen et al., 2023; Zhang et al., 2023).

Fungi can degrade organic matter such as lignin and cellulose, which are difficult for bacteria to decompose in the soil. Certain beneficial bacteria can recycle organic matter and mineral nutrients in the soil and promote nutrient transfer, which affects soil nutrient transformation and plant nutrient acquisition (Sui et al., 2019; Zhang WW. et al., 2019). In this study, at the phylum level, the dominant phylum Ascomycetes in the N2 treatment was significantly higher than that in the N0 treatment, which is consistent with the results of a previous study in which a high soil nitrogen level enhanced the dominance position of Ascomycota (Zhou et al., 2016). Other studies have shown that most of Ascomycota belong to saprophytes, and their relative abundance changes are related to soil organic matter content, and the significant changes in this study are consistent with the changes in OM content. It has also been reported that Basidiomycota has a stronger ability to decompose refractory substances such as lignin and cellulose (Osono, 2020). However, certain harmful Basidiomycota, such as powdery mildew and rust, cause smut and rust in crops, ultimately causing crop yield loss (Zhao et al., 2023). In this study, the relative abundances of Ascomycota and Basidiomycota increased with increasing of nitrogen addition, indicating that nitrogen addition increased the abundance of soil fungal populations and decreased the abundance of the most beneficial bacterial populations. This, in turn, disrupted the rhizosphere microbial balance and promoted the transformation of soil from bacterial soil to unhealthy fungal soil.

At the genus level, the *Rhodanobacter* population has also been shown to have antagonistic effects against the root rot fungal pathogen *Fusarium solani* and is involved in the Den process of the soil nitrogen cycle (Yang et al., 2021). *Bradyrhizobium* and *Mesorhizobium* are symbiotic nitrogen fixing bacteria that play important roles in soil organic matter decomposition and soil nutrient cycling, etc. *Phenylobacterium* has the effects of NF, and phosphorus dissolution and is beneficial to the mineralization of organic matter, *Microbacterium* is also a typical beneficial bacterium (Berendsen et al., 2018; Zhang et al., 2021). *Sphingomonas* and *Novosphingobium* are gram-negative bacteria widely distributed in nature, and can fix nitrogen in the soil nitrogen cycle. The metabolic mechanism of *Sphingomonas* makes it tolerant to poor nutrients; therefore it is extremely vigorous in nature (Pang et al., 2021; Liu et al., 2023). This was also confirmed by the highest content of *Sphingomonas* in the low nutrient N0 treatment in this study.

The above results were further verified using the LEfSe differential analysis of the microbial community in the rhizosphere soil of ginseng from phylum to species. A large number of Proteobacteria, Acidobacteria, Verrucomicrobia and Bacteroidetes were enriched in the N0 treatment, among which *Bradyrhizobium* and *Sphingomonas* have an important role in promoting plant quality formation. These bacteria also have very good potential for nitrogen fixation and biological control of antagonistic pathogenic bacteria and play an important role in maintaining the balance of ginseng rhizosphere microecology and nutrients (Kruczyńska et al., 2023). In contrast, enrichment in the N1 treatment *Trinickia* has potential plant pathogenicity (Li P. et al., 2022), suggesting that the enrichment of harmful microorganisms in plants is at the expense of beneficial microorganisms in plants. However, the enrichment of *Rhodanobacter* in N2 treatment was a well-known beneficial bacterium of plants, which has an antagonistic effect on the root rot fungal pathogen *Fusarium solani*. This indicates that ginseng will produce a “cry for help” strategy when it encounters external environmental stimuli and significantly recruit some beneficial bacteria to inhibit pathogenic fungi to maintain the normal growth of plants, which has certain development potential. However most pathogenic bacteria were present in *Mycolicibacterium*, and *Alcaligenaceae* was involved in the Den process and was a typical denitrifying bacterium, second only to *Pseudomonas* in its universality (Li P. et al., 2022). Therefore, the difference in the enrichment of beneficial microorganisms and pathogenic microorganisms in plants under different nitrogen levels may be another important indicator of the microbial driving changes in ginseng rhizosphere soil microecology affecting the yield and quality of ginseng.

**Figure 5:**
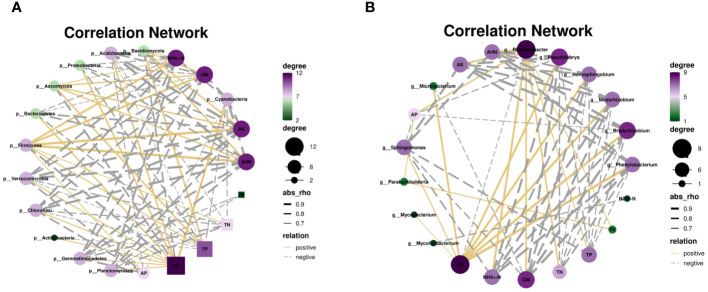
Correlation network between microbial composition and soil properties, phyla **(A)** and genus **(B)**.

Several studies have reported that various factors affect soil microbial communities’ compositions (Wu et al., 2024). The results of network analysis showed that some key rhizosphere microorganisms, such as Proteobacteria and Actinobacteria were significantly positively correlated with soil pH. Firmicutes, Basidiomycota and Ascomycota were significantly negatively correlated with pH (**Figure 5A** and **Supplementary Table S7**). *Sphingomonas* and *Bradyrhizobium* were significantly positively correlated with soil pH (**Figure 5B**). These results suggested that soil pH is an important factor driving changes in soil microbial diversity and community structure, which is consistent with previous reports (Zhou et al., 2018; Li et al., 2020; Yang et al., 2021).

In summary, an increase in nitrogen concentration reduces the number of beneficial microorganisms in the soil and increases the number of pathogenic microorganisms, which improves the occurrence of ginseng diseases. However when the nitrogen concentration exceeds a certain threshold, some beneficial bacteria that can resist environmental disturbance begin to increase to maintain the balance of soil microecology and enable plants to grow normally (Huang et al., 2015; Fudjoe et al., 2023).

Previous studies have focused on the abundance of individual functional genes related to NF, Nit and Den in the soil nitrogen cycle, such as *nifH*, *amoA*, *nirK*, and *nirS*, whereas other genes and process related genes have received less attention (Wang et al., 2021; Yang et al., 2024). In this study, we comprehensively evaluated the abundance of key functional genes in each process of soil nitrogen cycling and found that nitrogen addition significantly affected on the abundance of genes related to Den, ANR and DNR in the rhizosphere soil of ginseng, but did not significantly affect Nit and NF (**Figure 3A**).

Among the genes involved in Nit, nitrogen addition did not significantly affect *amoA*, *amoB*, *amoC* and hao genes, but significantly increased the abundance of *nxrA* genes, and *nxrB* genes showed no significant difference but also showed an upward trend. This finding differs from those of previous studies (Wang H. et al., 2023; Yang et al., 2018), which may be due to differences in the environments of the different basal soils. Furthermore Mantel analysis showed that the abundance of Nit genes was significantly positively correlated with the contents of TN and NH_4_^+^-N in the soil (*p <*0.05) (**Supplementary Table S8**), indicating that nitrogen content is one of the main causes of changes in Nit genes (Li Y. et al., 2022). Notably, the abundance of *amoA*, *amoB*, *amoC* and *hao* genes in the key gene *nxrA* and *nxrB* of NO_2_^-^ to NO_3_^-^ was higher than that of NH_3_^+^ to NO_2_^-^ (**Figures 3C, D**), indicating that nitrogen addition had little effect on the rate-limiting step ammonia oxidation process of the Nit reaction (Cai et al., 2020), which is consistent with the total Nit gene abundance results. However, *nxrA* and *nxrB* gene abundance may also promote Nit (Han et al., 2018). In addition, *nifH* is often used as a genetic marker for the detection of nitrogen fixing microorganisms in soil (Kuypers et al., 2018). In this study, the gene abundances of *nifH* and *nifD* were lower at different nitrogen levels, which was consistent with the low abundance of soil NF genes in *Robinia pseudoacacia* L (Ku et al., 2022). Among the genes associated with Den, we found that the *norB* and *nirK* genes were the most abundant, followed by the *nosZ*, *narI/J*, *napA/B*, *norC*, and *nirS* genes, which was inconsistent with the reports of Li (Li J. et al., 2022). It has also been found that the order of Den gene abundance is consistent with the order of the Den process (Chen et al., 2012); this difference may be related to the substrate differences in the Den process in different soil environments. In the nitrogen addition treatment, we found that N1 and N2 treatments significantly increased the abundance of *napA* gene (**Figure 3D**) (Liao et al., 2024), but did not significantly affect other genes. Previous studies have shown that genes related to the first two steps are more susceptible to nutrient changes, especially changes in nitrate concentration. During Den reduction, the nitrate concentration decreased due to plant uptake, leaching, and NO and N_2_O release, which may weaken the effect of nitrogen addition on the abundance of functional genes in the subsequent nitrogen cycling process (Klawonn et al., 2015; Tang et al., 2016). On the one hand, this result is influenced by the sequence of Den steps, and on the other hand, it has been proposed that *nosZ* gene has greater adaptability in various soils and is less affected by environmental factors than other genes (Zhong et al., 2014). Our inconsistent results may reflect the fact that changes in substrate concentrations under nitrogen addition did not significantly affect on the stimulation of *narG*, *nirK*, and *nirS* genes, which is similar to the results of the Li study (Li BB. et al., 2021). The abundance of Den genes was significantly positively correlated with the contents of TN, NH_4_^+^-N, AHN, TK and OM (p <0.01) (**Supplementary Table S8**), and the results reported in other studies are similar (Yang et al., 2023). The positive response of genes involved in Den to nitrogen addition may be due to an increase in rhizosphere unstable carbon substrates in support of heterotrophic Den. Several studies have shown that *norB* and *nirK* significantly promote soil N_2_O emissions (Pester et al., 2011). Although there was no significant difference between the two genes in this study, their relative abundance were high, and both showed an upward trend with the increase of nitrogen concentration. These results suggest that nitrogen addition increases the abundance of all genes involved in the Den process and promotes the soil nitrogen cycling process, which may lead to higher rates of soil nitrogen loss and greenhouse gas emissions. This study is important for understanding how nitrogen influences changes in the microbial community and the functional genes of ginseng rhizosphere soil. Our research focused on the impact of nitrogen application during the first year of cultivation, especially during the harvest season of ginseng. Given the complexity of farmland ecosystems, and the influence of variable environmental conditions on the soil microbial community, in the future, we need to conduct more detailed and long-term research. This will help us to better understand the driving forces and mechanisms that influence soil microbial community diversity, structure, and nitrogen cycling functional genes in farmland ecosystems under nitrogen application, ultimately informing sustainable agricultural practices.

## Conclusions

In conclusion, nitrogen can affect ginseng yield by changing the soil microbial community structure and the nitrogen cycling process. The results of this study showed that appropriate nitrogen addition (20 N g/m^2^) could significantly improve soil nutrient content and increase ginseng yield. However, when nitrogen was excessive (40 N g/m^2^), it significantly reduced soil NO_3_^–^N and pH, which in turn affected the diversity and composition of microbial communities, especially by reducing the abundance of beneficial bacteria and increasing the number of pathogenic microorganisms. In addition, N2 treatment significantly increased the total abundance of Den, ANR and DNR genes, and promoted the soil nitrogen cycling process, ultimately decreasing the nitrogen supply capacity and NO_3_^–^N content in the soil, thereby reducing ginseng yield. This study clarified the process of nitrogen transformation in the rhizosphere soil of ginseng and the response of functional microorganisms to nitrogen addition, providing a theoretical basis for further research on the sustainable development of ginseng agroecosystems.

## Data availability statement

The datasets presented in this study can be found in online repositories. The names of the repository/repositories and accession number(s) can be found below: https://www.ncbi.nlm.nih.gov/, PRJNA1094890.

## Author contributions

KL: Data curation, Methodology, Software, Writing – original draft, Writing – review & editing. HL: Conceptualization, Supervision, Writing – review & editing. MH: Conceptualization, Supervision, Writing – review & editing. LY: Conceptualization, Funding acquisition, Supervision, Writing – review & editing.
