# Supplementary material for: Soil metagenomics reveals the effect of nitrogen on soil microbial communities and nitrogen-cycle functional genes in the rhizosphere of Panax ginseng
Source: Front Plant Sci. 2024 Aug 7;15:1411073. doi: 10.3389/fpls.2024.1411073 (PMC11335670; doi:10.3389/fpls.2024.1411073)
Supplement: Supplementary file 1 [file DataSheet_1.docx]

Supplementary Material

Soil metagenomics reveals the effect of nitrogen on soil microbial communities and nitrogen-cycle functional genes in the rhizosphere of Panax ginseng

**Kexin Li^1^ , Hongmei Lin^1^*, Mei Han^1^*, Limin Yang^1^**

**^1^College of Traditional Chinese Medicine, Jilin Agricultural University, Changchun 130000, China;**

**e-mail: likexin@mails.jlau.edu.cn (K.L.); yanglimin@jlau.edu.cn (L.Y.)**

*** Correspondence: hongmeil@jlau.edu.cn (H.L.); meih@jlau.edu.cn (M.H.)**

**Supplementary Figures and Tables**


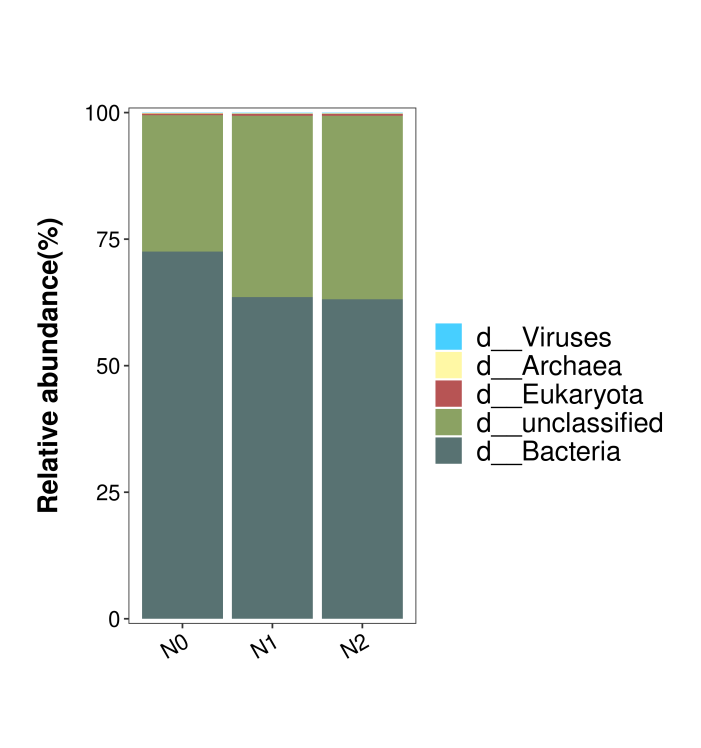


**Fig. S1** Relative abundance of soil microbial taxa under different nitrogen levels.


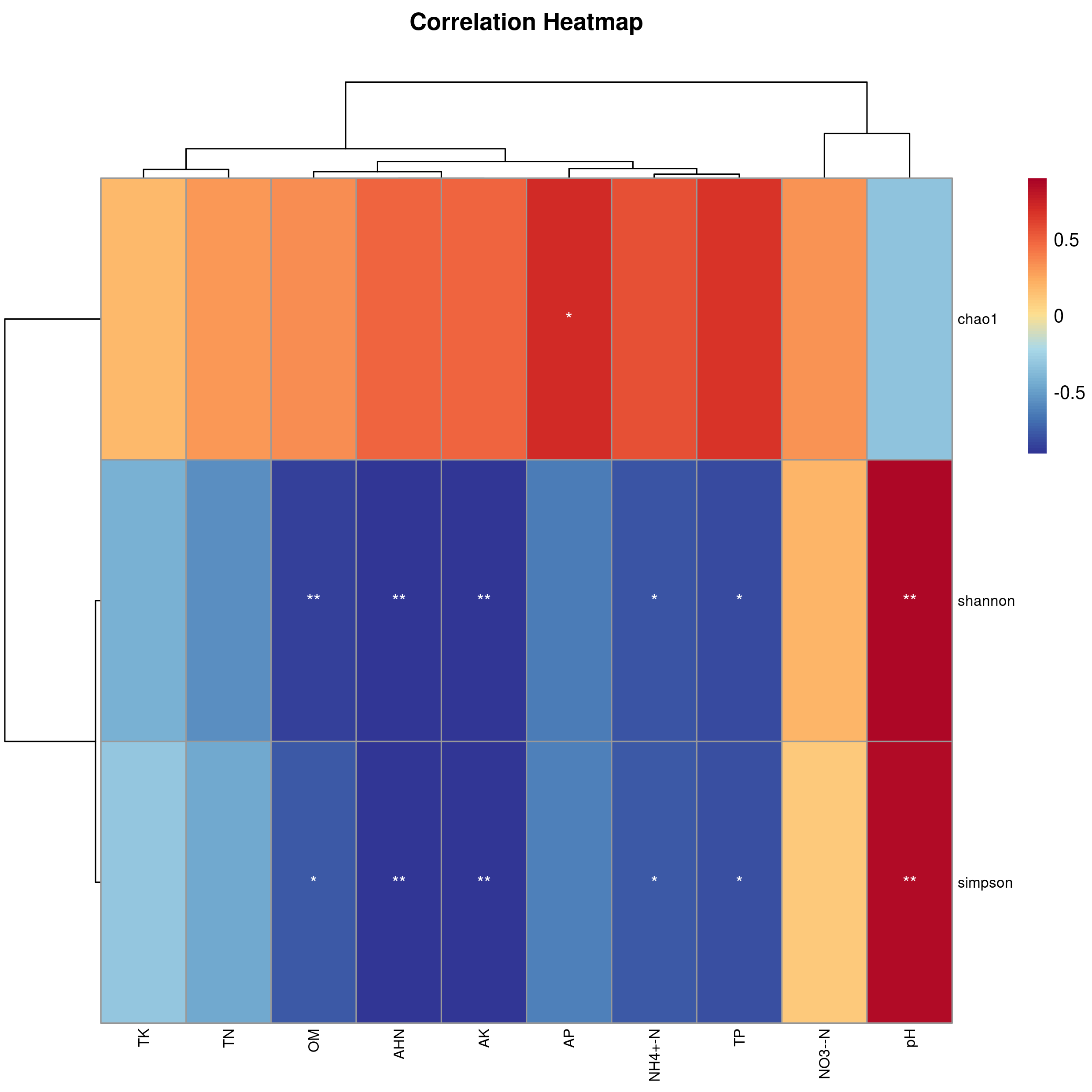


**Fig. S2** Relationship between rhizosphere soil properties and microbial diversity of ginseng (*p <0.05,**p <0.01).


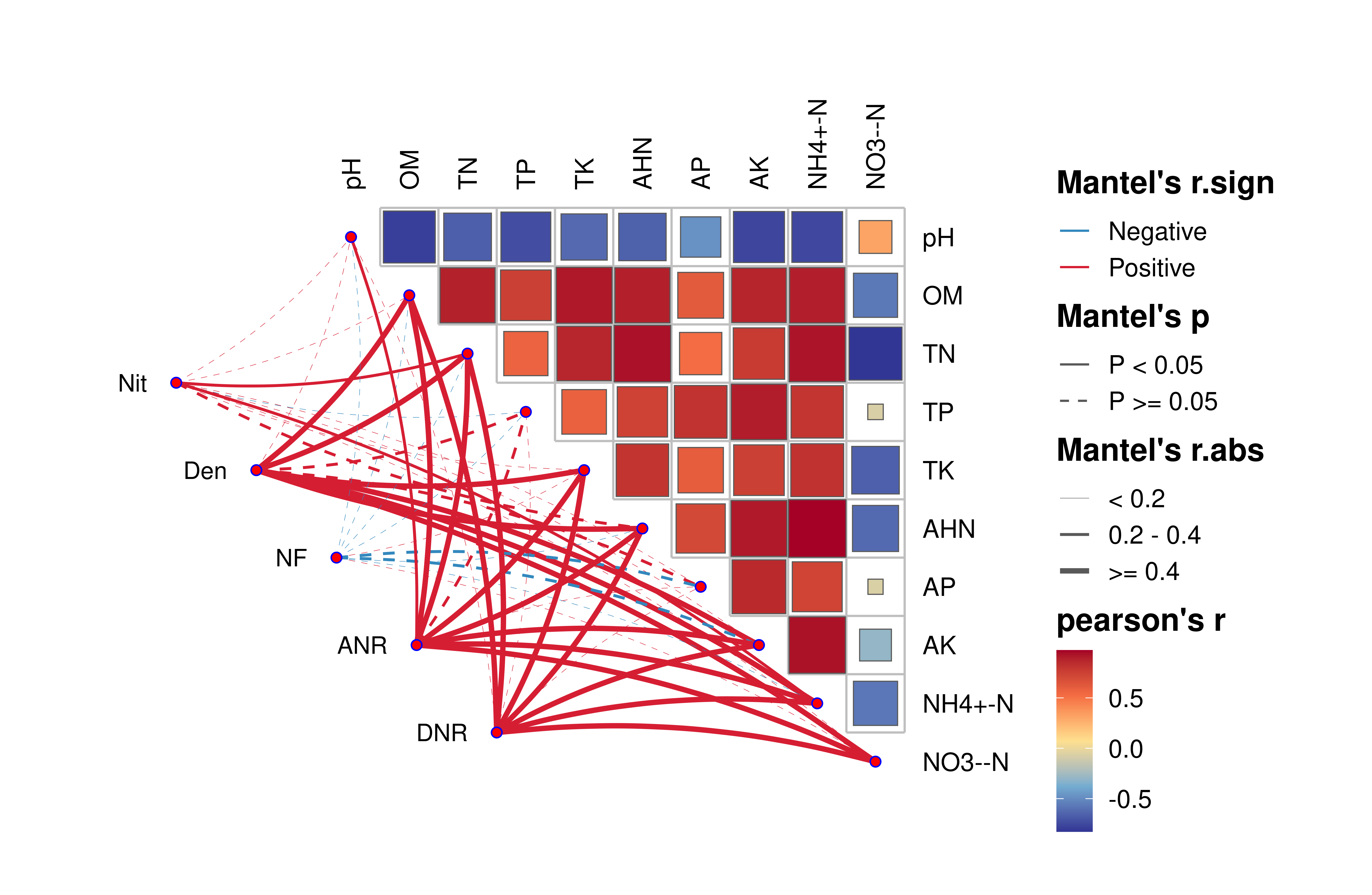


**Fig. S3** Relationship between functional genes of N cycle pathway and soil properties under different nitrogen levels(A), (p <0.05).

**Fig. S4** Expression of nitrogen cycling genes and TPM in ginseng rhizosphere soil.

**Table S1.** Overview of the soil metagenomic sequencing and annotation results of ginseng rhizosphere soil at different nitrogen levels

| Sample | Raw Data | Clean Tags | Contigs | N50（bp） | N75（bp） |
| --- | --- | --- | --- | --- | --- |
| N0_1 | 70289272 | 67747070 | 340141 | 883 | 637 |
| N0_2 | 69246758 | 67158440 | 302276 | 925 | 649 |
| N0_3 | 68640256 | 66391724 | 314259 | 895 | 643 |
| N1_1 | 69725624 | 67141890 | 321115 | 961 | 648 |
| N1_2 | 68920466 | 66455346 | 333626 | 902 | 637 |
| N1_3 | 70827250 | 68224852 | 337941 | 935 | 641 |
| N2_1 | 70001242 | 67438968 | 351628 | 925 | 638 |
| N2_2 | 63667584 | 61219020 | 312481 | 942 | 640 |
| N2_3 | 71063376 | 68657620 | 347170 | 936 | 640 |

N50 and N75 represent the length of the contig overlapping the midpoint and seventy-five percent of the length-order concatenation of contigs, respectively.

**Table S2.** Information of microbial functional genes involved in the nitrogen (N) cycling processes identified in this study. KO: Kyoto Encyclopedia of Genes and Genomes; EC: Enzyme Classification.

| KEGG orthology number [KO] | Gene name | Encoded protein [EC] |
| --- | --- | --- |
| KO: K10944 | *amoA* | ammonia monooxygenase subunit A/methane [EC:1.14.99.39,1.14.18.3] |
| KO: K10945 | *amoB* | ammonia monooxygenase subunit B/methane |
| KO: K10946 | *amoC* | ammonia monooxygenase subunit C/methane |
| KO: K10535 | *hao* | hydroxylamine dehydrogenase/Hydroxylamine oxidoreductase [EC:1.7.2.6, 1.7.2.8] |
| KO: K00370 | *narG, narZ, nxrA* | nitrate reductase/nitrite oxidoreductase, alpha subunit [EC:1.7.5.1 1.7.99.-] |
| KO: K00371 | *narH, narY, nxrB* | nitrate reductase/nitrite oxidoreductase, beta subunit [EC:1.7.5.1 1.7.99.-] |
| KO: K00374 | *narI* | nitrate reductase gamma subunit [EC:1.7.5.1 1.7.99.4] |
| KO: K00373 | *narJ* | nitrate reductase delta subunit |
| KO: K02567 | *napA* | periplasmic nitrate reductase [EC:1.9.6.1] |
| KO: K02568 | *napB* | periplasmic nitrate reductase, electron transfer subunit |
| KO: K00368 | *nirK* | nitrite reductase (NO-forming) [EC:1.7.2.1] |
| KO: K15864 | *nirS* | nitrite reductase (NO-forming) [EC:1.7.2.1 1.7.99.1] |
| KO: K04561 | *norB* | nitric oxide reductase subunit B [EC:1.7.2.5] |
| KO: K02305 | *norC* | nitric oxide reductase subunit C |
| KO: K00376 | *nosZ* | nitrous-oxide reductase [EC:1.7.2.4] |
| KO: K00372 | *nasA* | assimilatory nitrate reductase catalytic subunit [EC:1.7.99.-] |
| KO: K00367 | *narB* | nitrate reductase [EC:1.7.7.2] |
| KO: K00366 | *nirA* | ferredoxin-nitrite reductase [EC:1.7.7.1] |
| KO: K15876 | *nrfH* | nitrite reductase complex |
| KO: K00363 | *nirD* | nitrite reductase (NAD(P)H) [EC:1.7.1.15] |
| KO: K00362 | *nirB* | nitrite reductase [NAD(P)H], large subunit [EC:1.7.1.15] |
| KO: K02586 | *nifD* | nitrogenase molybdenum-iron protein alpha chain [EC:[1.18.6.1](https://www.genome.jp/entry/1.18.6.1)] |
| KO: K02588 | *nifH* | Nitrogenase subunit NifH (ATPase) [EC:1.18.6.1] |
| KO: K01428 | *ureC* | Urease subunit alpha [EC:3.5.1.5] |
| KO: K00261 | *gdhA* | glutamate dehydrogenase [EC:1.4.1.3] |
| KO: K00284 | *gltB* | Glutamate synthase [NADPH] large chain (NADPH-GOGAT) [EC:1.4.7.1] |
| KO: K00266 | *gltD* | Glutamate synthase [NADPH] small chain [EC:1.4.1.13 1.4.1.14] |
| KO: K01915 | *glnA* | Glutamine synthetase, type I, N-terminal (Glutamate--ammonia ligase I) (GSI) [EC:6.3.1.2] |
| KO: K15576 | *nrtA* | nitrate/nitrite ABC transporter substrate-binding protein |
| KO: K15577 | *nrtB* | nitrate/nitrite ABC transporter membrane protein |
| KO: K15578 | *nrtC* | nitrate ABC transporter ATP-binding protein [EC:3.6.3.-] |

**Table S3.** Effects of nitrogen on the number and diversity index of soil microbial species

| Treatments | Observed species | Chao1 | Shannon | Simpson | Goods coverage |
| --- | --- | --- | --- | --- | --- |
| N0 | 18344.33±37.54a | 18587.18±10.04b | 8.47±0.04a | 0.98±0.00a | 1.00±0.00a |
| N1 | 18404.33±19.60a | 18674.99±33.27a | 7.97±0.11b | 0.96±0.01b | 1.00±0.00a |
| N2 | 18371.67±29.37a | 18649.56±26.20a | 7.87±0.04b | 0.96±0.00b | 1.00±0.00a |

**Table S4.** Annotation of microbial taxa with different nitrogen treatments(%)

| Treatments | Observed species | Bacteria | Eukaryota | Archaea | Viruses |
| --- | --- | --- | --- | --- | --- |
| N0 | 18344.33±37.54a | 72.55±1.31a | 0.29±0.02b | 0.12±0.01a | 0.10±0.01b |
| N1 | 18404.33±19.60a | 63.55±2.70b | 0.38±0.03a | 0.13±0.02a | 0.12±0.01a |
| N2 | 18371.67±29.37a | 63.12±1.10b | 0.39±0.01a | 0.11±0.01a | 0.12±0.01a |

**Table S5.** Abundance of functional genes of N cycle pathway under different nitrogen levels

| Treatments | N0 | N1 | N2 | Significance |
| --- | --- | --- | --- | --- |
| ureC | 103.75±0.56 | 113.63±5.43 | 153.75±2.43 | yes |
| gdhA | 78.43±3.01 | 85.35±4.44 | 108.74±0.53 | yes |
| gltB | 671.45±28.12 | 698.39±17.91 | 874.96±19.85 | no |
| gltD | 222.25±10.10 | 242.60±12.68 | 302.33±14.71 | yes |
| glnA | 564.96±4.04 | 586.73±20.20 | 740.97±10.58 | no |
| nrtA | 36.06±0.86 | 38.16±0.71 | 63.70±0.93 | no |
| nrtB | 27.26±0.50 | 26.98±0.55 | 44.91±2.94 | no |
| nrtC | 34.88±1.02 | 38.99±2.09 | 59.89±1.58 | yes |

**Table S6.** The primer sets for amplification of functional genes involved in nitrogen cycle.

| Gene | Primer | Function | Refererce |
| --- | --- | --- | --- |
| amoA-B | amoB-1Fmod: CTGGGGTTTCTACTGGTGGTC | Nitrification | Meinhardt et al. 2015 |
|  | GenAOBR:GCAGTGATCATCCAGTTGCG |  |  |
| nxrA | nxrA1F: CAGACCGACGTGTGCGAAAG | Nitrification | Wertz et al. 2008 |
|  | nxrA2R: TCYACAAGGAACGGAAGGTC |  |  |
| napA | napAV17mf: TGGACVATGGGYTTYAAYC | Denitrification | Bru et al. 2007 |
|  | napA4r: ACYTCRCGHGCVGTRCCRCA |  |  |
| nirK | nirK876: ATYGGCGGVAYGGCGA | Denitrification | Henry et al. 2004 |
|  | nirK1040: GCCTCGATCAGRTTRTGGTT |  |  |
| norB | cnorB2F: GACAAGNNNTACTGGTGGT | Denitrification | Fang et al. 2018 |
|  | cnorB6R: GAANCCCCANACNCCNGC |  |  |
| nosZ | nosZ-F: CG(C/T) TGT TC(A/C) TCG ACA GCCAG | Denitrification | Throbäck et al. 2004 |
|  | NosZ-R: CGC (G/A)A(C/G) GGC AA(G/C) AAG GT(G/C) CG |  |  |
| nifH | nifH-F: ACCCGCCTGATCCTGCACGCCAAGG | Nitrogen fixation | Roesch et al. 2010 |
|  | nifH-R: ACGATGTAGATTTCCTGGGCCTTGTT |  |  |

**Table S7.** Relationship between functional genes of nitrogen cycle pathway and soil properties under different nitrogen levels

| spec | env | r | p | pd |
| --- | --- | --- | --- | --- |
| Nit | pH | 0.168308767 | 0.147 | P >= 0.01 |
| Nit | OM | 0.176570118 | 0.143 | P >= 0.01 |
| Nit | TN | 0.372097892 | 0.026 | P >= 0.01 |
| Nit | TP | -0.08385849 | 0.617 | P >= 0.01 |
| Nit | TK | 0.10327242 | 0.267 | P >= 0.01 |
| Nit | AHN | 0.274135119 | 0.061 | P >= 0.01 |
| Nit | AP | 0.064645693 | 0.314 | P >= 0.01 |
| Nit | AK | 0.158929864 | 0.155 | P >= 0.01 |
| Nit | NH_4_^+^-N | 0.332696256 | 0.032 | P >= 0.01 |
| Nit | NO_3_^-^-N | 0.015671315 | 0.441 | P >= 0.01 |
| Den | pH | 0.156442646 | 0.156 | P >= 0.01 |
| Den | OM | 0.749502328 | 0.003 | P < 0.01 |
| Den | TN | 0.851612308 | 0.002 | P < 0.01 |
| Den | TP | 0.251215436 | 0.077 | P >= 0.01 |
| Den | TK | 0.793778541 | 0.001 | P < 0.01 |
| Den | AHN | 0.806571214 | 0.004 | P < 0.01 |
| Den | AP | 0.252528902 | 0.073 | P >= 0.01 |
| Den | AK | 0.540482515 | 0.018 | P >= 0.01 |
| Den | NH_4_^+^-N | 0.845733229 | 0.002 | P < 0.01 |
| Den | NO_3_^-^-N | 0.476521148 | 0.03 | P >= 0.01 |
| NF | pH | -0.070374162 | 0.48 | P >= 0.01 |
| NF | OM | -0.114990834 | 0.72 | P >= 0.01 |
| NF | TN | -0.002706504 | 0.439 | P >= 0.01 |
| NF | TP | -0.044835803 | 0.516 | P >= 0.01 |
| NF | TK | 0.032575426 | 0.368 | P >= 0.01 |
| NF | AHN | -0.10464347 | 0.694 | P >= 0.01 |
| NF | AP | -0.303473141 | 0.984 | P >= 0.01 |
| NF | AK | -0.215724058 | 0.913 | P >= 0.01 |
| NF | NH_4_^+^-N | -0.102872925 | 0.718 | P >= 0.01 |
| NF | NO_3_^-^-N | 0.035379711 | 0.337 | P >= 0.01 |
| ANR | pH | 0.390111133 | 0.009 | P < 0.01 |
| ANR | OM | 0.818335005 | 0.003 | P < 0.01 |
| ANR | TN | 0.857067339 | 0.001 | P < 0.01 |
| ANR | TP | 0.244923999 | 0.08 | P >= 0.01 |
| ANR | TK | 0.875497409 | 0.001 | P < 0.01 |
| ANR | AHN | 0.737423306 | 0.005 | P < 0.01 |
| ANR | AP | 0.190255826 | 0.116 | P >= 0.01 |
| ANR | AK | 0.48853656 | 0.023 | P >= 0.01 |
| ANR | NH_4_^+^-N | 0.797733938 | 0.003 | P < 0.01 |
| ANR | NO_3_^-^-N | 0.476712283 | 0.029 | P >= 0.01 |
| DNR | pH | 0.146801197 | 0.167 | P >= 0.01 |
| DNR | OM | 0.646276712 | 0.009 | P < 0.01 |
| DNR | TN | 0.890761115 | 0.003 | P < 0.01 |
| DNR | TP | 0.174762345 | 0.144 | P >= 0.01 |
| DNR | TK | 0.700360876 | 0.006 | P < 0.01 |
| DNR | AHN | 0.814057425 | 0.003 | P < 0.01 |
| DNR | AP | 0.162043468 | 0.159 | P >= 0.01 |
| DNR | AK | 0.452844358 | 0.019 | P >= 0.01 |
| DNR | NH_4_^+^-N | 0.83607259 | 0.003 | P < 0.01 |
| DNR | NO_3_^-^-N | 0.580703949 | 0.018 | P >= 0.01 |

**Table S8.** Relationship between microbe and soil properties under different nitrogen levels

| Data1 | Data2 | rho | pvalue | relation |
| --- | --- | --- | --- | --- |
| AHN | p__Firmicutes | 0.97 | 0.000165344 | positive |
| AK | p__Firmicutes | 0.97 | 0.000165344 | positive |
| p__Cyanobacteria | pH | 0.93 | 0.000309097 | positive |
| OM | p__Firmicutes | 0.90 | 0.000827411 | positive |
| NH_4_^+^-N | p__Firmicutes | 0.90 | 0.002028219 | positive |
| p__Basidiomycota | TP | 0.87 | 0.004508377 | positive |
| p__Acidobacteria | pH | 0.86 | 0.00295628 | positive |
| p__Proteobacteria | pH | 0.85 | 0.003601934 | positive |
| AHN | p__Basidiomycota | 0.85 | 0.006073633 | positive |
| AK | p__Basidiomycota | 0.85 | 0.006073633 | positive |
| p__Ascomycota | TP | 0.85 | 0.006073633 | positive |
| p__Bacteroidetes | pH | 0.83 | 0.006108555 | positive |
| AHN | p__Ascomycota | 0.82 | 0.0107694 | positive |
| AK | p__Ascomycota | 0.82 | 0.0107694 | positive |
| OM | p__Ascomycota | 0.81 | 0.007889298 | positive |
| OM | p__Basidiomycota | 0.79 | 0.01044452 | positive |
| p__Firmicutes | TP | 0.78 | 0.01722332 | positive |
| p__Verrucomicrobia | pH | 0.78 | 0.01255343 | positive |
| p__Chloroflexi | pH | 0.78 | 0.01255343 | positive |
| p__Actinobacteria | pH | 0.76 | 0.01804078 | positive |
| p__Gemmatimonadetes | pH | 0.75 | 0.02017365 | positive |
| p__Planctomycetes | pH | 0.75 | 0.02017365 | positive |
| p__Firmicutes | TN | 0.73 | 0.03112324 | positive |
| AP | p__Firmicutes | 0.73 | 0.03112324 | positive |
| NH_4_^+^-N | p__Ascomycota | 0.73 | 0.03112324 | positive |
| NH_4_^+^-N | p__Basidiomycota | 0.72 | 0.03686618 | positive |
| AHN | p__Acidobacteria | -0.98 | 4.96032E-05 | negtive |
| AK | p__Acidobacteria | -0.98 | 4.96032E-05 | negtive |
| NH_4_^+^-N | p__Acidobacteria | -0.97 | 0.000165344 | negtive |
| AHN | p__Cyanobacteria | -0.97 | 0.000165344 | negtive |
| AK | p__Cyanobacteria | -0.97 | 0.000165344 | negtive |
| OM | p__Cyanobacteria | -0.96 | 3.28586E-05 | negtive |
| AHN | p__Verrucomicrobia | -0.95 | 0.000352734 | negtive |
| AK | p__Verrucomicrobia | -0.95 | 0.000352734 | negtive |
| AHN | p__Chloroflexi | -0.95 | 0.000352734 | negtive |
| AK | p__Chloroflexi | -0.95 | 0.000352734 | negtive |
| NH_4_^+^-N | p__Verrucomicrobia | -0.92 | 0.001311728 | negtive |
| AHN | p__Gemmatimonadetes | -0.92 | 0.001311728 | negtive |
| AK | p__Gemmatimonadetes | -0.92 | 0.001311728 | negtive |
| NH_4_^+^-N | p__Chloroflexi | -0.92 | 0.001311728 | negtive |
| NH_4_^+^-N | p__Planctomycetes | -0.92 | 0.001311728 | negtive |
| NH_4_^+^-N | p__Gemmatimonadetes | -0.90 | 0.002028219 | negtive |
| AHN | p__Planctomycetes | -0.90 | 0.002028219 | negtive |
| AK | p__Planctomycetes | -0.90 | 0.002028219 | negtive |
| OM | p__Gemmatimonadetes | -0.90 | 0.001098526 | negtive |
| p__Firmicutes | pH | -0.89 | 0.001156085 | negtive |
| p__Ascomycota | pH | -0.89 | 0.001501313 | negtive |
| AHN | p__Bacteroidetes | -0.88 | 0.003075397 | negtive |
| AK | p__Bacteroidetes | -0.88 | 0.003075397 | negtive |
| AP | p__Gemmatimonadetes | -0.88 | 0.003075397 | negtive |
| NH_4_^+^-N | p__Cyanobacteria | -0.88 | 0.003075397 | negtive |
| OM | p__Verrucomicrobia | -0.88 | 0.001815796 | negtive |
| OM | p__Chloroflexi | -0.88 | 0.001815796 | negtive |
| OM | p__Planctomycetes | -0.87 | 0.002273778 | negtive |
| AP | p__Verrucomicrobia | -0.87 | 0.004508377 | negtive |
| AP | p__Chloroflexi | -0.87 | 0.004508377 | negtive |
| OM | p__Acidobacteria | -0.85 | 0.003418215 | negtive |
| p__Basidiomycota | pH | -0.85 | 0.003601934 | negtive |
| p__Gemmatimonadetes | TN | -0.85 | 0.006073633 | negtive |
| p__Planctomycetes | TN | -0.85 | 0.006073633 | negtive |
| AHN | p__Proteobacteria | -0.83 | 0.008267196 | negtive |
| AK | p__Proteobacteria | -0.83 | 0.008267196 | negtive |
| p__Proteobacteria | TP | -0.82 | 0.0107694 | negtive |
| p__Bacteroidetes | TP | -0.82 | 0.0107694 | negtive |
| AP | p__Planctomycetes | -0.82 | 0.0107694 | negtive |
| p__Verrucomicrobia | TN | -0.78 | 0.01722332 | negtive |
| p__Chloroflexi | TN | -0.78 | 0.01722332 | negtive |
| p__Cyanobacteria | TP | -0.78 | 0.01722332 | negtive |
| OM | p__Proteobacteria | -0.77 | 0.01524018 | negtive |
| NH_4_^+^-N | p__Proteobacteria | -0.77 | 0.02138999 | negtive |
| NH_4_^+^-N | p__Bacteroidetes | -0.77 | 0.02138999 | negtive |
| AP | p__Acidobacteria | -0.77 | 0.02138999 | negtive |
| p__Gemmatimonadetes | TK | -0.77 | 0.02138999 | negtive |
| p__Cyanobacteria | TN | -0.77 | 0.02138999 | negtive |
| p__Planctomycetes | TK | -0.75 | 0.02549052 | negtive |
| p__Acidobacteria | TN | -0.73 | 0.03112324 | negtive |
| p__Actinobacteria | TP | -0.72 | 0.03686618 | negtive |
| p__Verrucomicrobia | TP | -0.72 | 0.03686618 | negtive |
| p__Chloroflexi | TP | -0.72 | 0.03686618 | negtive |
| OM | p__Bacteroidetes | -0.71 | 0.03165656 | negtive |
| p__Acidobacteria | TP | -0.70 | 0.04325397 | negtive |
| AP | p__Cyanobacteria | -0.70 | 0.04325397 | negtive |
